# Robust CPD Algorithm for Non-Rigid Point Set Registration Based on Structure Information

**DOI:** 10.1371/journal.pone.0148483

**Published:** 2016-02-11

**Authors:** Lei Peng, Guangyao Li, Mang Xiao, Li Xie

**Affiliations:** 1 College of Electronics and Information Engineering, Tongji University, Shanghai, China; 2 College of Information Engineering, Taishan Medical University, Taian, Shandong, China; University of North Carolina, UNITED STATES

## Abstract

Recently, the Coherent Point Drift (CPD) algorithm has become a very popular and efficient method for point set registration. However, this method does not take into consideration the neighborhood structure information of points to find the correspondence and requires a manual assignment of the outlier ratio. Therefore, CPD is not robust for large degrees of degradation. In this paper, an improved method is proposed to overcome the two limitations of CPD. A structure descriptor, such as shape context, is used to perform the auxiliary calculation of the correspondence, and the proportion of each GMM component is adjusted by the similarity. The outlier ratio is formulated in the EM framework so that it can be automatically calculated and optimized iteratively. The experimental results on both synthetic data and real data demonstrate that the proposed method described here is more robust to deformation, noise, occlusion, and outliers than CPD and other state-of-the-art algorithms.

## Introduction

Point set registration is one of the main methods for image registration. As a fundamental component of the computer vision field, point set registration is often used in medical image processing [1–3], pattern recognition [4], and remote sensing image processing [5, 6]. Generally, point set registration is divided into either rigid or non-rigid registration. Rigid registration is a relatively simple process that mainly processes the scaling, translation, and rotation of the point sets. Generalized rigid registration includes affine and projection transformation. However, for non-rigid registration, the transformation form is non-rigid, and it is difficult to accurately determine the transformation model, especially if there is a large degree of degradation, such as deformation, occlusion, noise, or outliers.

Non-rigid point set registration is faced with two problems: one is finding the correspondence between two point sets, and the other is choosing the right transformation model. If one of these problems is solved, then the other can be solved easily. However, the issue is that both of the problems are often unknown and must be solved simultaneously. Therefore, most algorithms use the iterative method of alternate calculations of correspondence and transformation to obtain an approximately optimal solution.

The iterative closest point (ICP) algorithm [7], which is one of the most famous point set registration algorithms, is mainly used for the registration of free curves and surfaces. The ICP algorithm assumes the closest points to be in correspondence and minimizes the mean square distance iteratively until the objective function converges. Instead of assigning a binary correspondence, soft assignment and relaxation labeling have been proposed for fuzzy correspondences. One of the most well-known contributions for these work is a non-rigid point matching generic framework proposed by Chui et al. [8]. Under this framework, the TPS-RPM algorithm uses a thin plate spline as non-rigid mapping as well as uses soft allocation and deterministic annealing to solve the combined optimization problem. The probabilistic methods [9–13] are typically used to solve the problem of point set registration. Myronenko et al. [9, 14] proposed the Coherent Point Drift (CPD) algorithm and treated the alignment of the two point sets as a probability density estimation problem. Furthermore, restrained by the motion coherence theory, the Gaussian Mixture Model (GMM) centroids fit to the data points via the EM algorithm. Many improvements to CPD have been proposed that involve altering some phases of this algorithm [15–20]. Jian et al. [10] used two GMMs to represent the point sets, and the differences of the two GMMs are minimized to solve the problem of point set registration. Moreover, there are some additional algorithms [21–25] that use other kinds of mixture model for point set registration. However, these methods mainly use the global relationships to find the correspondence and hardly consider the structure information.

To make full use of the neighborhood structure information of the point sets, Belongie et al. [4] introduced the Shape Context (SC) to measure the similarity between two shapes, and the corresponding points with a similar shape will have a similar SC. Furthermore, SC is used in shape matching and object recognition. Zheng et al. [26] introduced the concept of neighborhood structure and proposed a robust non-rigid point matching algorithm by keeping the neighborhood structure. They used the SC distance to find the initial correspondence, followed by a relaxation labeling technique to search for the optimal solution. Ma et al. [27] and Chen et al. [28] used the descriptor of SC to first establish the rough correspondence and then used the robust L_2_E estimator to estimate the transformation. In addition, different structure descriptor [29–34] of the point sets are used for point set registration. Although these algorithms use the structure information as the similarity measurement, they find the correspondence and the transformation separately. In this paper, we propose a GMM based method and use neighborhood structure information combining global distance to determine the correspondence, jointly, refine the transformation in the EM process.

As a rapid method that can be used in high dimensional point set registration, the CPD algorithm is relatively advantageous. However, the method is limited in the following two main aspects. (1) Only the distance is considered at the step of computing similarity, not the neighborhood structure of points, and each GMM component has an equal contribution to the distribution of data points without making full use of the similarity, resulting in sensitivity to noise, occlusion and outliers. (2) The outlier ratio must be manually assigned, but the exact value of the outlier ratio is often impossible to determine before registration. In addition, CPD uses an improper uniform distribution to model noise and outliers so that even if the real outlier ratio is set, it still cannot guarantee a good result.

In this paper, we devise an improvement based on the state-of-the-art CPD algorithm to solve the aforementioned problem. The main contributions of our paper are twofold. (1) Structure information is used to perform the auxiliary calculation of the correspondence. Here, the shape context is used as the structure descriptor of the points to compute the similarity, and the proportion of each GMM component is adjusted by the similarity; as a result, the registration process is more accurate and robust. (2) The automatic estimation of the outlier ratio is integrated into the EM framework so that the product of the outlier ratio and the uniform distribution can “capture” noise and outliers properly. This process allows us to implement this method without manually selecting the real outlier ratio, thus improving its practicability.

The rest of this paper is organized as follows: Section 2 reviews the CPD algorithm, followed by our proposed method to improve CPD with detailed description of the two aspects of the structure descriptor and the processing outliers in the EM framework. Section 3 gives the experimental results and performance evaluation on synthetic data and real data. Finally, the discussion and conclusion are presented in section 4.

## Methods

### 1. Review of the Coherent Point Drift algorithm

#### 1.1 Coherent Point Drift algorithm

The CPD algorithm considers the alignment of two point sets as a probability density estimation problem. One point set represents the GMM centroids, and the other point set represents the data points. At the optimum, the correspondence of the two point sets is obtained by maximizing the posterior probability. The core of CPD is to force the GMM centroids to move coherently as a group to preserve the topological structure of the point sets.

Given two point sets *X* and *Y*. The points in the “model” set *Y* = {*y*_*m*_|1, 2, …, *M*} are considered GMM centroids. The points in the “scene” set *X* = {*x*_*n*_|1, 2, …, *N*} are considered data points generated by the GMM. The purpose is to learn a transformation *T* applied to *Y* for *X* = *T*(*Y*, *θ*), in which *θ* is a set of the transformation parameters. The probability density function of the GMM can be written as follows:

$$p\left(x\right)=\sum_{m=1}^{M+1}P\left(m\right)p\left(x|m\right)$$ (1)

where $p\left(x|m\right)=\frac{1}{{\left(2\pi \sigma^{2}\right)}^{D/2}}exp\left(-\frac{∥x-T\left(y_{m},\theta \right){∥}^{2}}{2\sigma^{2}}\right)$, *m* = 1, 2, …, *M*. To handle the outliers, occlusion, and noise, a uniform distribution is used: *p*(*x*|*M*+1) = 1/*N*. The weight of the uniform distribution, which represents the outlier ratio, is denoted as *ω*, 0 ≤ *ω* ≤ 1. In addition, an equal isotropic covariance *σ*^2^ and equal membership probabilities *P*(*m*) = (1 − *ω*)/*M* are used for all GMM components. Thus, the probability density function takes the following form:

$$p\left(x\right)=\omega \frac{1}{N}+\left(1-\omega \right)\frac{1}{M}\sum_{m=1}^{M}p\left(x|m\right)$$ (2)

The centroid of the GMM is parameterized by the set of parameters *θ*. The parameters will be estimated by maximizing the likelihood, which minimizes the negative log-likelihood function:

$$E\left(\theta ,\sigma^{2}\right)=-\sum_{n=1}^{N}log\sum_{m=1}^{M+1}P\left(m\right)p\left(x_{n}|m\right)$$ (3)

According to Jensen’s inequality, an upper bound of the negative log-likelihood function in Eq (3) is the function of *Q* in Eq (4), which is the objective function of CPD.

$$Q=-\sum_{n=1}^{N}\sum_{m=1}^{M+1}P^{old}\left(m|x_{n}\right)log\left[P\left(m\right)p\left(x_{n}|m\right)\right]$$ (4)

Assuming that each Gaussian component is independent, the correspondence probability between two points, *y*_*m*_ and *x*_*n*_, is defined as the posterior probability of the GMM centroid given the data point: *P*(*m*|*x*_*n*_) = *P*(*m*)*p*(*x*_*n*_|*m*)/*p*(*x*_*n*_). Next, the EM framework is used to solve the optimal estimate of *θ* and *σ*^2^. The CPD algorithm iteratively computes the posterior probability distribution *P*^*old*^(*m*|*x*_*n*_) of mixture components by the Bayes’ theorem in E-step and determines the new parameter values by minimizing the expectation of the complete negative log-likelihood function in M-step until convergence. The registration process of CPD algorithm is given as follows.

Initialization: *ω*, *θ*, and *σ*^2^ are initialized.

E-step: First, the old values of the parameters are guessed, and then the posterior probability distribution *P*^*old*^(*m*|*x*_*n*_) of the mixture components is calculated:

$$\begin{array}{c} P^{old}\left(m|x_{n}\right)=\frac{exp(-\frac{∥x_{n}-T\left(y_{m},\theta \right){∥}^{2}}{2\sigma^{2}})}{\sum_{k=1}^{M}exp\left(-\frac{∥x_{n}-T\left(y_{k},\theta \right){∥}^{2}}{2\sigma^{2}}\right)+\frac{\omega M{\left(2\pi \sigma^{2}\right)}^{D/2}}{(1-\omega )N}},\;\left(m=1,2,...,M\right) \end{array}$$ (5)

M-step: The new parameters *θ* and *σ*^2^ are computed by minimizing the expectation of the complete negative log-likelihood function *Q*.

$$\begin{array}{c} Q\left(\theta ,\sigma^{2}\right)=\frac{1}{2\sigma^{2}}\sum_{n=1}^{N}\sum_{m=1}^{M}P^{old}\left(m|x_{n}\right){∥x_{n}-T\left(y_{m},\theta \right)∥}^{2}+\frac{DN_{P}}{2}log\sigma^{2} \end{array}$$ (6)

where $N_{P}=\sum_{n=1}^{N}\sum_{m=1}^{M}P^{old}\left(m|x_{n}\right)$.

The algorithm iterates E-step and M-step until *Q* converges. Next, the aligned point set is *T*(*Y*, *θ*).

#### 1.2 The limitations of CPD

From the discussion above, we can see that the outlier ratio *ω* and the Gaussian membership probabilities *P*(*m*) play significant roles in determining the distribution (one of the GMM components or the uniform distribution) of the data points. However, these parameters are fixed in CPD, so there are two problems that influence its performance severely. The first problem is that the similarity measure only depends on the Euclidean distance between points. In the case of a large degree of degradation, such as deformation, noise, occlusion, or outliers, the closest point pairs may not be in correspondence, whereas the point pairs that have the similar neighbor structures are probably in correspondence. Therefore, the contribution of each Gaussian component to the GMM is not the same. The neighborhood structure similarity of the point pairs should be introduced into the proportion of the GMM components.

The other problem existing in CPD is the outlier ratio *ω*, which requires manual assignment during initialization. This requirement also limits the application of CPD. It is difficult to determine the outlier ratio of the two point sets before registration. An improper value of *ω* leads to an unpredictable registration result. Meanwhile, CPD uses a uniform distribution 1/*N* to treat noise and outliers. The uniform distribution should be related to the coordinate range of the data points rather than the number of data points *N*. For example, given two data sets with the same number of points, the point’s distribution range of one data set is larger than the other. Thus, the uniform distribution of the two data sets should not be equal. As a result, even if the real outlier ratio is assigned, a good result is still not guaranteed. Only by slight adjustment of *ω* to make the product of *ω* and 1/*N* model the noise and outliers appropriately. Therefore, in the CPD algorithm, *ω* can only be called an approximate outlier ratio.

### 2. The method of improvement

The paper proposes a method to overcome the two limitations of CPD stated above. As previously mentioned, the CPD algorithm does not take into account the structure information of the points. Generally, two corresponding points also have a similar neighborhood structure, which allows us to compute the similarity and find the correspondence according to the neighborhood structure information. Because the contribution of each Gaussian component is not the same, the proportion of each GMM component is adjusted by the similarity. In addition, because the outlier ratio must be manually assigned in CPD, we formulate it in the EM framework so that it can be automatically calculated and optimized iteratively.

#### 2.1 Structure descriptor

In this paper, shape context [4] is used as a structure descriptor. The shape context at a point is the measurement of the distribution of other points relative to it. This point is treated as the origin of the log-polar coordinate, and the direction from this point to the mass center of the shape is used as the positive x-axis. Thus, the shape context has translation, scale, and rotation invariant characteristics. In considering two points, *x*_*n*_ is in one set and *y*_*m*_ is in another set; their shape contents are *h*_*n*_(*k*) and *h*_*m*_(*k*), respectively, where *h*(*k*) is the value of k-bin for the log-polar histogram, *k* = 1, 2, …, *K*. Let *C*_*nm*_ represent the matching cost measure of these two points.

$$C_{nm}=exp\left(-\frac{1}{2\gamma }\sum_{k=1}^{K}\frac{{\left[h_{n}\left(k\right)-h_{m}\left(k\right)\right]}^{2}}{h_{n}\left(k\right)+h_{m}\left(k\right)}\right),\;\left(\gamma =0.1\;in\;default\right)$$ (7)

The more similar the shape contexts for the two points of *x*_*n*_ and *y*_*m*_ are, the more likely it is that the correspondence and the membership probability *P*_*n*_(*m*) are higher. Thus, the membership probabilities are improved as *P*_*n*_(*m*) = (1 − *ω*)*C*_*nm*_/*S*, where $S=\sum_{k=1}^{M}C_{nk}$ and the probability density function of GMM is rewritten as

$$p\left(x_{n}\right)=\omega \frac{1}{N}+\left(1-\omega \right)\frac{1}{S}\sum_{m=1}^{M}C_{nm}p\left(x_{n}|m\right)$$ (8)

After introducing the structure descriptor to the registration process, at the first several times of iterations, the similarity measure based on structure descriptor increases the speed of finding the correct correspondences. However, at the later stage of the iterations, most of the correspondences are found. Thus, for the similarity measure, the effect of structure descriptor is reduced, and the effect of Euclidean distance is increased.

#### 2.2 Outliers processing and the EM solution

In the CPD algorithm, the outlier ratio *ω* must be manually assigned. However, it is difficult to determine the real outlier ratio in advance. If an incorrect *ω* value is assigned, then the performance of the registration will be reduced. Here, an automatically calculated outlier ratio is considered as well as an iterative process of the EM algorithm.

The non-rigid point set registration method proposed in this paper uses the Tikhonov regularization framework. The transformation is defined as the initial position plus a displacement function *v*: *T*(*Y*, *v*) = *Y*+*v*(*Y*). In the Reproducing Kernel Hilbert Space (RKHS), the regularization term is defined as *λ*‖*Lv*‖^2^/2. As the objective function of our method, by ignoring the constants, the negative log likelihood function Eq (6) can be written as

$$\begin{array}{cc} Q(v,\omega ,\sigma^{2})= & \frac{1}{2\sigma^{2}}\sum_{n=1}^{N}\sum_{m=1}^{M}P^{old}\left(m|x_{n}\right){∥x_{n}-y_{m}-v\left(y_{m}\right)∥}^{2}+\frac{DN_{P}}{2}log\sigma^{2}\\ & -N_{P}log\left(1-\omega \right)-\left(N-N_{P}\right)log\omega +\frac{\lambda }{2}{∥Lv∥}^{2} \end{array}$$ (9)

where the set of unknown parameters is {*v*, *ω*, *σ*^2^}. The integrated stages for solving the parameters by the EM algorithm are given as follows.

E-step: The posterior probability *P*^*old*^(*m*|*x*_*n*_) can be calculated by applying Bayes rule:

$$\begin{array}{cc} P^{old}\left(m|x_{n}\right)= & \frac{C_{nm}exp\left(-\frac{∥x_{n}-y_{m}-v\left(y_{m}\right){∥}^{2}}{2\sigma^{2}}\right)}{\sum_{k=1}^{M}C_{nk}exp\left(-\frac{∥x_{n}-y_{k}-v\left(y_{k}\right){∥}^{2}}{2\sigma^{2}}\right)+\frac{\omega S{\left(2\pi \sigma^{2}\right)}^{D/2}}{(1-\omega )N}},\;\left(m=1,2,...,M\right) \end{array}$$ (10)

Denote *P* = (*P*_*mn*_)_*M*×*N*_, where *P*_*mn*_ = *P*^*old*^(*m*|*x*_*n*_) is a soft decision, which indicates what degree point *y*_*m*_ corresponds to point *x*_*n*_. The probability of noise and outliers can be computed as follows:

$$P^{old}\left(M+1|x_{n}\right)=1-\sum_{m=1}^{M}P^{old}\left(m|x_{n}\right)$$ (11)

M-step: The new parameters *v*, *ω*, and *σ*^2^ can be calculated by minimizing the objective function *Q*. *σ*^2^ is updated as follows:

$$\sigma^{2}=\frac{1}{DN_{P}}\sum_{n=1}^{N}\sum_{m=1}^{M}P\left(m|x_{n}\right){∥x_{n}-y_{m}-v\left(y_{m}\right)∥}^{2}$$ (12)

Next, the function *Q*(*v*, *ω*, *σ*^2^) is minimized with respect to the outlier ratio to obtain the value of *ω*.

$$\omega =1-\frac{1}{N}\sum_{n=1}^{N}\sum_{m=1}^{M}P\left(m|x_{n}\right)$$ (13)

which equals $\omega =\frac{1}{N}\sum_{n=1}^{N}P\left(M+1|x_{n}\right)$. However, when using Eq (13) to update *ω* directly, it is problematic if the outlier ratio is large because over-fitting will occur. Therefore, to avoid this problem, the outlier ratio can be updated by using the following function:

$$\omega =\omega^{old}+\alpha \left(\omega^{new}-\omega^{old}\right),\;\left(\alpha =\frac{1}{t}\;in\;default\right)$$ (14)

where *ω*^*old*^ is the old value of *ω*, which is updated at the last iteration. *ω*^*new*^ is calculated by Eq (13). *α* is the learning ratio, which is similar to the annealing rate in the simulated annealing algorithm; it is an empirical parameter to avoid over-learning. In this paper, the learning ratio is set as *α* = 1/*t*, where *t* is the current iteration time. The learning ratio is larger at the beginning and then gradually become smaller so that *ω* can update more rapidly at the initial iterations to achieve rapid optimization and update slower at the later iterations to prevent over-fitting. Thus, the outlier ratio can be efficiently updated, combined with the learning rate, during the EM iteration by Eqs (13) and (14).

The functional form of *v*, which must be solved by calculus of variation, must satisfy the Euler-Lagrange differential equation. By solving the differential equation, the displacement function *v* can be obtained. Here, a Gaussian matrix kernel is used to define the hypothesis space *H*.

$$\left[G+\lambda \sigma^{2}diag{\left(P1\right)}^{-1}\right]W=diag{\left(P1\right)}^{-1}PX-Y$$ (15)

where *W*_*M*×*D*_ = (*W*_1_, ..,*W*_*M*_) is a matrix of coefficients, *G* = (*y*_*i*_, *y*_*j*_)_*M*×*M*_ is the kernel matrix with elements *G*(*y*_*i*_, *y*_*j*_) = *exp*(−‖*y*_*i*_−*y*_*j*_‖^2^/2*β*), *diag*()^−1^ is the inverse diagonal matrix, and *P*1 is the matrix of posterior probability *P* multiplied by the column vector of all ones.

The algorithm iterates E-step and M-step until *Q* converges. Next, the transformed positions of *y*_*m*_ are determined by *T*(*Y*, *W*) = *Y* + *GW*, and the probability of correspondence is given by *P*.

In the expression *T*(*Y*, *v*) = *Y* + (*Y*), *Y* is a constant, and *v*(*Y*) is a regularization; both are differentiable so that the sum of them is still differentiable. However, the partial differential equation *v*(*Y*), which must be solved by calculus of variation, may obtain an infinite number of solutions under different boundary conditions. Therefore, the transformation *T* is not reversible and is not diffeomorphic.

## Results

The performance of our proposed method is tested with both synthetic data and real data. All experiments are performed by MATLAB R2013a on a PC with 16 GB of RAM and an i7-4770K (3.5 GHz) Inter CPU. In this section, the goal of the point set registration is to align the model point set onto the scene point set, where the model point set is presented by blue pluses and the scene point set is red circles.

### 1. Evaluation criterions

The registration error of two point sets is quantified as the average Euclidean distance between a point in the model set and the ground truth corresponding point in the scene set. Root Mean Square Error (RMSE) is used to measure the registration error.

$$Error_{p}=\sqrt{\frac{1}{J}\sum_{j=1}^{J}{\left(x_{j}-y_{j}\right)}^{2}}$$ (16)

where *J* is the total number of the ground truth correspondences, and *y*_*j*_ is the true corresponding point to *x*_*j*_.

The registration error of two images is defined as RMSE, which reflects the difference of intensity distribution of the two images.

$$Error_{i}=\sqrt{\frac{1}{U\times V}\sum_{u=1}^{U}\sum_{v=1}^{V}{\left[M\left(u,v\right)-F\left(u,v\right)\right]}^{2}}$$ (17)

where *U*×*V* is the total number of the pixels in each image, *M*(*u*, *v*) and *F*(*u*, *v*) are the intensity values of the pixels located in (*u*, *v*) of the two respective images.

Recall, precision, and *F*_1_ measure are well known parameters in statistics and pattern recognition. The recall is defined as the proportion of true-positive correspondences to the ground truth correspondences. The recall-accuracy curve, as used in [10, 25], denotes the ability of a registration method to determine as many true-positive correspondences as possible with low errors in accuracy. The *F*_1_ measure is used to evaluate the balance between recall and precision.

$$Recall=\frac{TP}{TP+FN}$$ (18)

$$Precision=\frac{TP}{TP+FP}$$ (19)

$$F_{1}=\frac{2\cdot Precision\cdot Recall}{Precision+Recall}=\frac{2\cdot TP}{2\cdot TP+FP+FN}$$ (20)

where *TP* denotes true-positive, *FN* denotes false-negative, and *FP* denotes false-positive.

### 2. Experiments on synthetic data

The same data as in [8, 26, 27], named Chui-Rangarajan synthesized data, were used, and the shape of the fish and the Chinese character were selected. Four models of data designed to measure the robustness of registration algorithms under deformation, noise, occlusion, and outliers were chosen. Each model contains 5 different degradation levels, and each level includes 100 examples. In the rest of this section, the experimental results of our algorithm are shown, and the performance is analyzed from two aspects: the “structure descriptor based vs. constant memberships” and the “automatic outlier ratio vs. fixed one”.

#### 2.1 Results on synthetic data

For the purpose of evaluating the performance of the proposed method, the results of our method are compared with four state-of-the-art algorithms: GMMREG [10], TPS-RPM [8], RPM-L2E [27], and CPD [9]. The TPS-RPM algorithm uses soft assignment to find the correspondence under the deterministic annealing scheme, and the GMMREG algorithm uses the L_2_ distance between two GMMs to measure similarity. Both of the two algorithms do not leverage the neighborhood structure information of points as well as the CPD algorithm. The RPM-L2E algorithm depends on only the shape context to find the correspondence and optimize it in the iteration. However, our method uses the structure descriptor combined with the Euclidean distance to find correspondence in an EM framework. Therefore, those four algorithms are selected to compare with our method.

**Fig 1:**
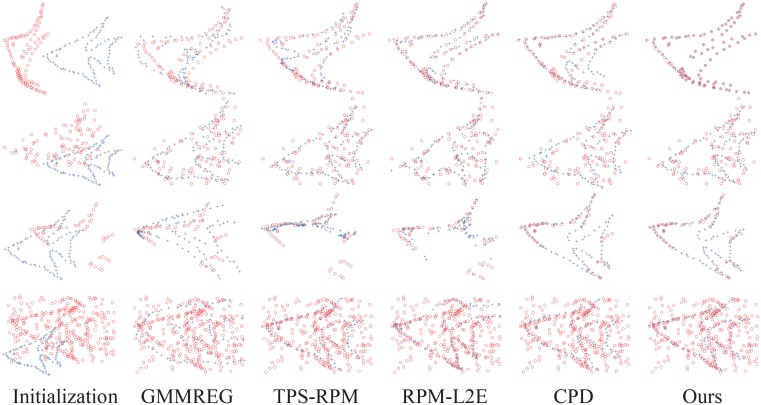
Registration examples on the fish point set. From top to bottom are the four largest degradations: deformation (0.08), noise (0.05), occlusion (0.5), and outlier (2.0). The goal is to align the model point set (blue pluses) onto the scene point set (red circles).

**Fig 2:**
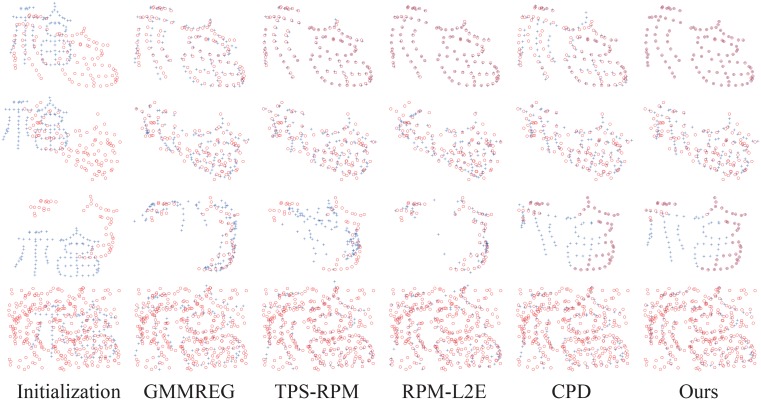
Registration examples on the Chinese character point set. From top to bottom are the four largest degradations: deformation (0.08), noise (0.05), occlusion (0.5), and outlier (2.0). The model point set is shown in blue pluses, and the scene point set is shown in red circles.

In each experiment, both the CPD algorithm and our method have the maximum iteration number set at 100, and the same initial values of the outlier ratio are assigned, as shown in Table 1. The registration examples are shown in Fig 1 (the fish shape) and Fig 2 (the Chinese character shape). In the two figures, from top to bottom, there are the four groups under the largest degradation levels of deformation (degree 0.08), noise (level 0.05), occlusion (ratio 0.5), and outlier (outlier-to-data ratio 2.0).

**Table 1:** Initial values of outlier ratio.

**Deformation**					
Degree of deformation	0.020	0.035	0.050	0.065	0.080
Initial *ω*	0	0	0	0	0
**Noise**					
Noise level	0.01	0.02	0.03	0.04	0.05
Initial *ω*	0.1	0.2	0.3	0.4	0.5
**Occlusion**					
Occlusion ratio	0.1	0.2	0.3	0.4	0.5
Initial *ω*	0.1	0.2	0.3	0.4	0.5
**Outlier**					
Outlier-to-data ratio	0.0	0.5	1.0	1.5	2.0
Initial *ω*	0	0.4	0.5	0.6	0.7

Both CPD and our method are set the same initial values of *ω* in each experiment

**Fig 3:**
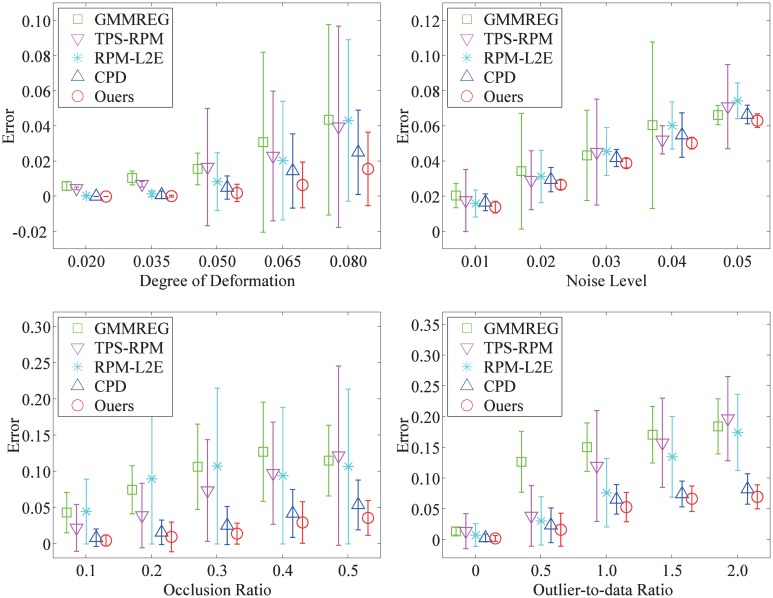
Performances of the registration methods on the fish point set. The registration performance of our method compared with the performances of GMMREG, TPS-RPM, RPM-L2E, and CPD on the fish point set. Each error bar indicates the registration error mean and the standard deviation over 100 trials.

**Fig 4:**
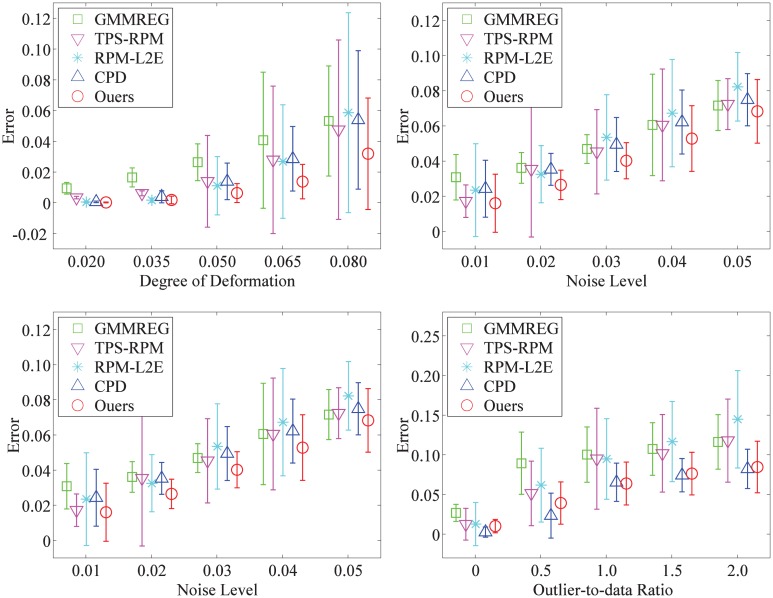
Performances of the registration methods on the Chinese character point set. The registration performances of GMMREG, TPS-RPM, RPM-L2E, CPD, and our method on the Chinese character point set. Each error bar indicates the registration error mean and the standard deviation over 100 trials.

The performance statistics for deformation, noise, occlusion, and outliers are summarized in Fig 3 (the fish shape) and Fig 4 (the Chinese character shape). Each algorithm is compared by the error mean and the standard deviation of the registration error of all 100 examples in each distortion degree. From the experimental and statistical results, we can see that our method is much more robust compared with the other four algorithms and generally give better performance, especially as the degradation degree increases.

#### 2.2 Evaluation of the structure descriptor

**Fig 5:**
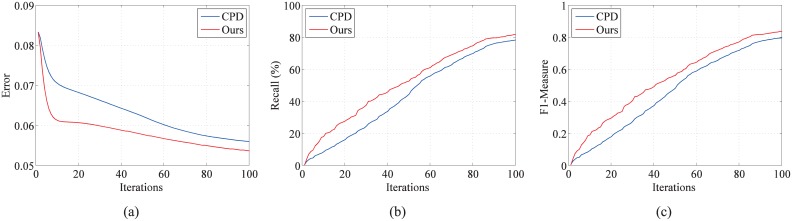
Evaluation of the effect of structure descriptor in the iteration. One hundred trials of the fish point set under the outlier degradation (outlier-to-data ratio 1.0) are chosen to test the effect of structure descriptor in the iteration: (a) error curves, (b) recall curves, and (c) *F*_1_ measure scores in the iteration. Our method is compared with CPD, both of which have the same initial value: *ω* is 0.7 and the maximum iteration number is 100.

To evaluate the effect of structure descriptor, the automatic outlier function is closed in the source code of our method and 100 trials of the fish point set under the outlier degradation (outlier-to-data ratio of 1.0) are chosen. Both CPD and our method are set the same initial values: *ω* is 0.7 and the maximum iteration number is 100. The error curves, the recall curves, and the *F*_1_ measure of the two algorithms during the iteration are shown in Fig 5. Here, the recall is defined as the proportion of the true-positive correspondences that are found at current iteration to the ground truth correspondences. As shown in the figure, due to the introduction of structure descriptor, at the first 20 times of the iterations, our method can find the correct correspondences rapidly and maintain smaller errors. At the later stage of the iterations, the effect of the structure descriptor is reduced and the effect of Euclidean distance is increased; as a result, the curves of the two algorithms tend to be similar.

However, the introduction of structure descriptor increases the complexity of the calculations. The average registration times of each example on the synthetic dataset are GMMREG: 0.24 s, TPS-RPM: 0.03 s, RPM-L2E: 6.52 s, CPD: 0.15 s, and our method: 1.21 s. The RPM-L2E algorithm and our method (which used shape context) exhibit poor speed performance.

#### 2.3 Evaluation of the automatic outlier processing

**Fig 6:**
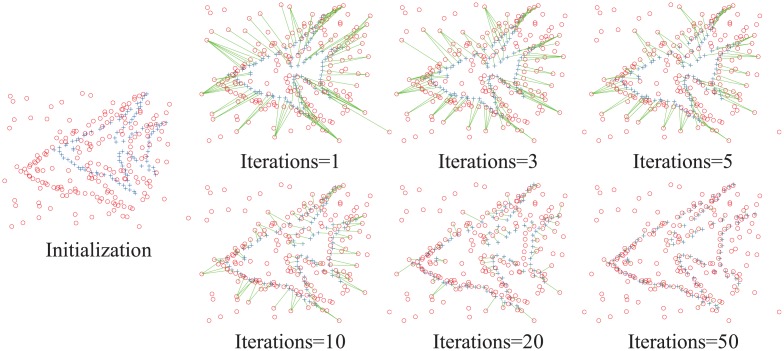
The change of the correspondence and the outliers in the iteration. The leftmost figure is the initial point sets with 98 points in the model point set (blue pluses) and 196 points in the scene point set (red circles). The right six figures are the correspondence and the distribution of the outliers in the iterations of 1, 3, 5, 10, 20 and 50 times of our method. The corresponding point pairs are connected by green lines, and the other points are the outliers.

In this subsection, the automatic outlier processing is evaluated in the EM framework. First, consider one example with 98 points in the model point set and 196 points in the scene point set to demonstrate the distribution of the outliers of our method during the iteration. The initial value of the outlier ratio is 0.7, and the maximum iteration number is 100. As shown in Fig 6, the leftmost figure is the initial point sets. The right six figures are the correspondence and the distribution of the outliers at the iterations of 1, 3, 5, 10, 20 and 50 times. The corresponding point pairs are connected by green lines. The many-to-one correspondence can be seen directly. There may be one Gaussian centroid closest to multiple date points and another Gaussian centroid that is not the closest to any data point. In other words, each point in the model point set has a closest point in the scene point set. However, some points in the scene point set cannot find the corresponding point to them; such points are so-called outliers. Whether a point is an outlier or not will change during the iteration of the EM framework. At the same time, we can see the change of the correspondence and the distribution of the outliers rapidly at the initial iterations, which then gradually slow and stabilize.

**Fig 7:**
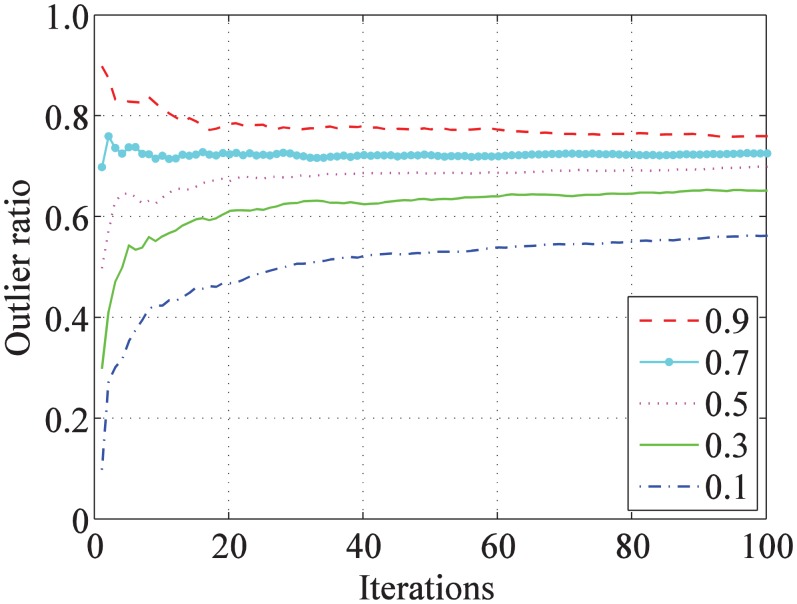
The statistics of the outlier ratio change in the iteration. The same point sets are assigned the different initial values of *ω* (0.1, 0.3, 0.5, 0.7, and 0.9). The outlier ratios converge to the optimal value iteratively.

The statistical figure of outlier ratio change in the iteration is shown in Fig 7. The learning ratio is set as *α* = 1/*t*, and the outlier ratios are, respectively, initialized as 0.1, 0.3, 0.5, 0.7, and 0.9. In each of the five experiments, the value of the outlier ratio converges to nearly 0.7 after iterating 100 times. On one hand, if the learning ratio is too small, then the outlier ratio does not converge to the optimal value at the end of iteration. On the other hand, if the learning ratio is too large, then the outlier ratio may converge to a local optimal solution.

**Fig 8:**
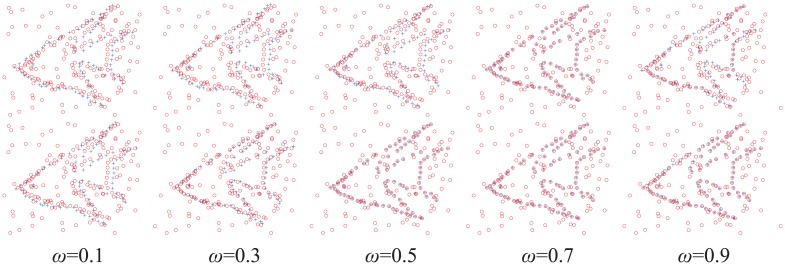
Registration examples with different initial outlier ratios. The same point sets are assigned different initial outlier ratios (0.1, 0.3, 0.5, 0.7, and 0.9). The top is the registration results of the CPD algorithm, and the bottom is our method.

**Fig 9:**
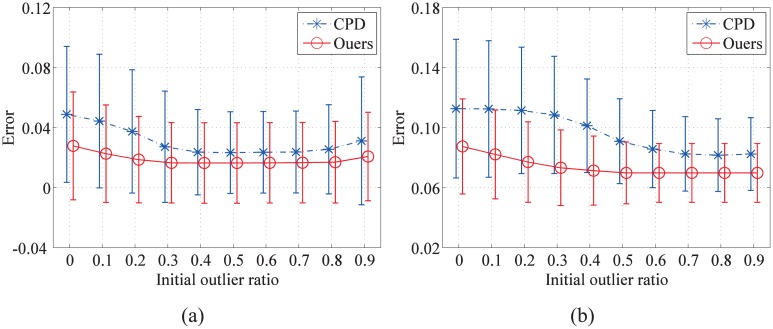
Performances of the registration methods for different initial outlier ratios. Two groups examples with outlier-to-data ratios of 0.5 (a) and 2.0 (b) are chosen to test the influence of different initial outlier ratios (from 0 to 0.9) on the registration results. The error means and the standard deviations of 100 examples in each group of our method are compared with CPD.

Furthermore, to evaluate the automatic outlier ratio of our method vs. the fixed one of the CPD algorithm, we assign different initial outlier ratios (0.1, 0.3, 0.5, 0.7, and 0.9) to the same example (the initial state is shown in Fig 6) and run the CPD algorithm together with our method. The registration results are shown in Fig 8. Next, two groups of the fish point set under the outlier degradation (outlier-to-data ratios of 0.5 and 2.0) are chosen to compare the influence of different initial outlier ratios on the registration results of CPD and our method. Both methods are assigned different initial outlier ratios from 0 to 0.9. The error means and the standard deviations of the registration error of 100 examples in each group are shown in Fig 9.

### 3. Experiments on real data

In this subsection, some real examples are presented on several biomedical datasets obtained from http://radiology.dxy.cn. The sizes of the original images are 240×180 pixels. The point sets are created by the edges of the objects, which are extracted using a regional scanning method. We demonstrate the registration results of all the experiments with the initial outlier ratio of 0.7 and the maximum iteration number of 100.

**Fig 10:**
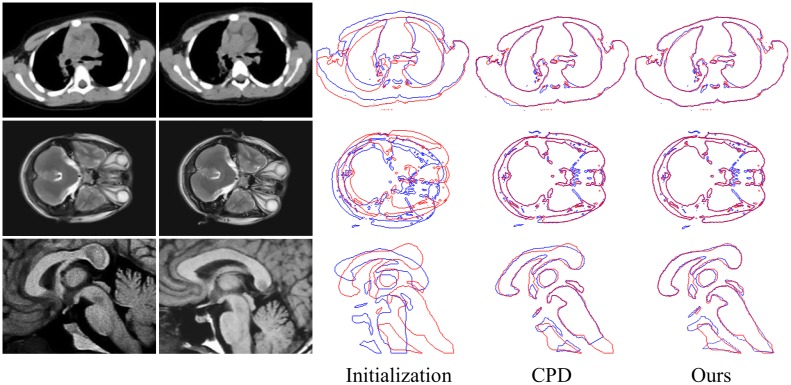
Registration examples on the real data. The top row is a pair of thorax CT images, the middle row is a pair of transverse plane from brain MRI, and the bottom row is a pair of sagittal plane from brain MRI. For each row, the leftmost two images are the scene data and the model data. The next image is the initial state of the point sets extracted from the scene data and the model data. The rightmost two images are the registration results using CPD and our method, in which the model point sets (blue) align to the scene point sets (red).

Three groups of the real data examples are shown in Fig 10. The top row is a pair of thorax Computed Tomography (CT) images. The middle row is a pair of transverse plane from brain Magnetic Resonance Imaging (MRI) data. The bottom row is the sagittal plane from brain MRI data, from which the boundaries of the apparatuses (corpus callosum, corpus fornicis, thalamus, basis pontis, and medulla oblongata) are extracted for registration. For each group, the leftmost two images are the scene data (fixed images) and the model data (moving images). The next image is the initial state of the point sets created by the scene data and the model data. The rightmost two images are the registration results by CPD and our method, in which the model point sets (blue) align to the scene point sets (red). Because of the limitation of the contour extraction algorithm and for reasons inherent to the original images, these point sets contain numerous outliers and noise. However, our method performs well and demonstrates visually accurate image alignments under deformation, outliers, and noise.

**Fig 11:**
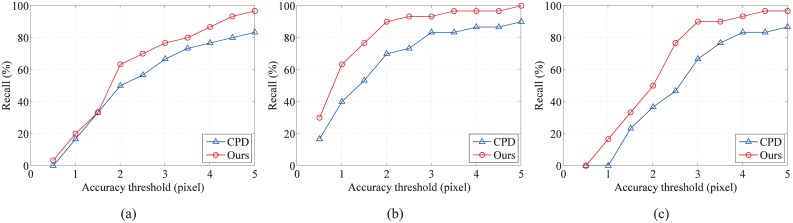
Performances of registration methods on the real data. Recall-accuracy curves are used to evaluate CPD and our method on the three real data experiments under the accuracy threshold 1 to 5 pixels: (a) thorax CT data, (b) transverse plane brain MRI data, and (c) sagittal plane brain MRI data.

To ensure a quantitative experimental comparison, we use the recall-accuracy curve as the evaluation criterion. First, for each pair of real data, we manually select 30 point pairs with obvious features as ground truth correspondences. Here, *TP* is defined as the number of the ground truth corresponding point pairs that fall within a given accuracy threshold in terms of pairwise distance. *FN* is defined as the number of the ground truth corresponding point pairs that fall outside the given accuracy threshold. The recall-accuracy curves of the three real data experiments, under different accuracy threshold (from 1 to 5 pixels), are shown in Fig 11. The proposed method can obtain better recall values in most of the tested accuracy thresholds than CPD.

**Fig 12:**
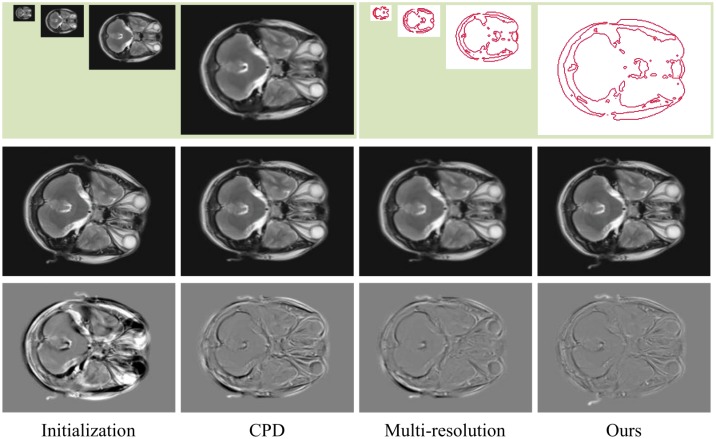
Registration example and comparison of the multi-resolution method. The top row shows the pyramid images and the corresponding contour point sets of the fixed image. The middle row, from left to right, shows the initial moving image and the registration results of CPD, multi-resolution, and our method. The bottom row shows the differences between the images of the middle row and the fixed image.

Because the structure descriptor provides some information of larger scale and a hierarchical registration framework can do the same, we compare the proposed method with the multi-resolution registration method. The pair of sagittal plane brain MRI data in Fig 10 is selected again, and a multi-resolution registration method is designed to perform the experiment. The conventional CPD algorithm is applied with multi-resolution strategy. As shown in Fig 12, we construct 4 level pyramid images. In each level, the CPD algorithm is used, and the registration result is used as the initial value of the next level. The registration error of the CPD algorithm, multi-resolution method, and our method are listed in Table 2. The experimental results show that the performance of the multi-resolution method has certain improvements compared with the conventional CPD algorithm but still no more than our method because the multi-resolution method requires down sampling, point set extraction, image interpolation, and transformation, etc. The error in each level transfers to the next level; thus, the cumulative error will inevitably increase. However, our method finds the optimal solution using only the structure descriptor and EM iteration processing. Thus, our method can avoid the accumulation of errors.

**Table 2:** Registration error for the sagittal plane brain MRI data.

	CPD	Multi-resolution	Our method
Error	18.0001	13.5107	8.2350

## Discussion

The paper presents a method that is an improvement upon the CPD algorithm. Our contribution consists of two aspects. First, adding structural information by means of the shape context to calculate the similarity and find the correspondence, and adjusting the proportion of each GMM component according to the similarity. Second, the outlier ratio is automatically calculated and optimized in the EM framework, thereby avoiding manual assignment of the wrong value (which may lead to an undesirable registration result). The experimental results show that our approach is robust to a large degree of degradation and outperforms CPD and other state-of-the-art non-rigid point set registration algorithms. Future work will involve further investigation of how to better use the structure information of point sets to find the correct correspondences in the presence of occlusion, noise, and outliers, in addition to studying how to increase the speed performance of the registration method that uses the structure descriptor.

## References

[1] Sotiras A, Davatzikos C, Paragios N. Deformable medical image registration: A survey. Medical Imaging, IEEE Transactions on. 2013;32(7):1153–1190. 10.1109/TMI.2013.2265603 PMC3745275 23739795

[2] Wang G, Wang Z, Chen Y, Zhao W. Robust point matching method for multimodal retinal image registration. Biomedical Signal Processing and Control. 2015;19:68–76. 10.1016/j.bspc.2015.03.004

[3] Han X. Feature-constrained nonlinear registration of lung CT images Medical image analysis for the clinic: a grand challenge. 2010; p. 63–72.

[4] Belongie S, Malik J, Puzicha J. Shape matching and object recognition using shape contexts. Pattern Analysis and Machine Intelligence, IEEE Transactions on. 2002;24(4):509–522. 10.1109/34.993558 16285381

[5] Zhang K, Li X, Zhang J. A robust point-matching algorithm for remote sensing image registration. Geoscience and Remote Sensing Letters, IEEE. 2014;11(2):469–473. 10.1109/LGRS.2013.2267771

[6] Wang S, You H, Fu K. BFSIFT: A novel method to find feature matches for SAR image registration. Geoscience and Remote Sensing Letters, IEEE. 2012;9(4):649–653. 10.1109/LGRS.2011.2177437

[7] Besl PJ, McKay ND. Method for registration of 3-D shapes In: Robotics-DL tentative. International Society for Optics and Photonics; 1992 p. 586–606.

[8] Chui H, Rangarajan A. A new point matching algorithm for non-rigid registration. Computer Vision and Image Understanding. 2003;89(2):114–141. 10.1016/S1077-3142(03)00009-2

[9] Myronenko A, Song X. Point set registration: Coherent point drift. Pattern Analysis and Machine Intelligence, IEEE Transactions on. 2010;32(12):2262–2275. 10.1109/TPAMI.2010.46 20975122

[10] Jian B, Vemuri BC. Robust point set registration using gaussian mixture models. Pattern Analysis and Machine Intelligence, IEEE Transactions on. 2011;33(8):1633–1645. 10.1109/TPAMI.2010.223 21173443

[11] Ma J, Chen J, Ming D, Tian J. A mixture model for robust point matching under multi-layer motion. PloS one. 2014;9(3):e92282 10.1371/journal.pone.0092282 24658087 PMC3962380

[12] Gerogiannis D, Nikou C, Likas A. Registering sets of points using Bayesian regression. Neurocomputing. 2012;89:122–133. 10.1016/j.neucom.2012.02.018

[13] Qu HB, Li JC, Wang JQ, Xiang L, Tao HJ. Robust Point Set Matching under Variational Bayesian Framework. In: Pattern Recognition (ICPR), 2014 22nd International Conference on. IEEE; 2014. p. 58–63.

[14] Myronenko A, Song X, Carreira-Perpinán MA. Non-rigid point set registration: Coherent point drift. In: Advances in Neural Information Processing Systems; 2006. p. 1009–1016.

[15] Hu Y, Rijkhorst EJ, Manber R, Hawkes D, Barratt D. Deformable vessel-based registration using landmark-guided coherent point drift In: Medical Imaging and Augmented Reality. Springer; 2010 p. 60–69.

[16] Wang P, Wang P, Qu Z, Gao Y, Shen Z. A refined coherent point drift (CPD) algorithm for point set registration. Science China Information Sciences. 2011;54(12):2639–2646. 10.1007/s11432-011-4465-7

[17] Habert S, Khurd P, Chefd’hotel C. Registration of multiple temporally related point sets using a novel variant of the coherent point drift algorithm: application to coronary tree matching In: SPIE Medical Imaging. International Society for Optics and Photonics; 2013 p. 86690M–86690M.

[18] Gao Y, Ma J, Zhao J, Tian J, Zhang D. A robust and outlier-adaptive method for non-rigid point registration. Pattern Analysis and Applications. 2014;17(2):379–388. 10.1007/s10044-013-0324-z

[19] Liu S, Sun G, Niu Z, Li N, Chen Z. Robust rigid coherent point drift algorithm based on outlier suppression and its application in image matching. Journal of Applied Remote Sensing. 2015;9(1):095085–095085. 10.1117/1.JRS.9.095085

[20] de Sousa S, Kropatsch WG. Graph-based point drift: Graph centrality on the registration of point-sets. Pattern Recognition. 2015;48(2):368–379. 10.1016/j.patcog.2014.06.011

[21] Sang Q, Zhang JZ, Yu Z. Robust non-rigid point registration based on feature-dependant finite mixture model. Pattern Recognition Letters. 2013;34(13):1557–1565. 10.1016/j.patrec.2013.06.019

[22] Tao W, Sun K. Asymmetrical Gauss Mixture Models for Point Sets Matching. In: Computer Vision and Pattern Recognition (CVPR), 2014 IEEE Conference on. IEEE; 2014. p. 1598–1605.

[23] Zhou Z, Zheng J, Dai Y, Zhou Z, Chen S. Robust non-rigid point set registration using student’s-t mixture model. PloS one. 2014;9(3):e91381 10.1371/journal.pone.0091381 24618749 PMC3950182

[24] Wang G, Wang Z, Zhao W, Zhou Q. Robust Point Matching Using Mixture of Asymmetric Gaussians for Nonrigid Transformation In: Computer Vision–ACCV 2014. Springer; 2014 p. 433–444.

[25] Wang G, Wang Z, Chen Y, Zhao W. A robust non-rigid point set registration method based on asymmetric gaussian representation. Computer Vision and Image Understanding. 2015;141:67–80. 10.1016/j.cviu.2015.05.014

[26] Zheng Y, Doermann D. Robust point matching for nonrigid shapes by preserving local neighborhood structures. Pattern Analysis and Machine Intelligence, IEEE Transactions on. 2006;28(4):643–649. 10.1109/TPAMI.2006.81 16566512

[27] Ma J, Zhao J, Tian J, Tu Z, Yuille AL. Robust estimation of nonrigid transformation for point set registration. In: Computer Vision and Pattern Recognition (CVPR), 2013 IEEE Conference on. IEEE; 2013. p. 2147–2154.

[28] Chen J, Ma J, Yang C, Ma L, Zheng S. Non-rigid point set registration via coherent spatial mapping. Signal Processing. 2015;106:62–72. 10.1016/j.sigpro.2014.07.004

[29] Lee JH, Won CH. Topology preserving relaxation labeling for nonrigid point matching. Pattern Analysis and Machine Intelligence, IEEE Transactions on. 2011;33(2):427–432. 10.1109/TPAMI.2010.179 20876932

[30] Yan Xw, Wang W, Zhao J, Hu Jm, Zhang J, Wan Jw. Relaxation labeling for non-rigid point matching under neighbor preserving. Journal of Central South University. 2013;20:3077–3084. 10.1007/s11771-013-1831-1

[31] Jin F, Feng D. Image Registration Algorithm Using Mexican Hat Function-Based Operator and Grouped Feature Matching Strategy. PloS one. 2014;9(4):e95576 10.1371/journal.pone.0095576 24752223 PMC3994077

[32] Ge S, Fan G, Ding M. Non-rigid Point Set Registration with Global-Local Topology Preservation. In: Computer Vision and Pattern Recognition Workshops (CVPRW), 2014 IEEE Conference on. IEEE; 2014. p. 245–251.

[33] Tang J, Shao L, Zhen X. Robust point pattern matching based on spectral context. Pattern Recognition. 2014;47(3):1469–1484. 10.1016/j.patcog.2013.09.017

[34] Yang Y, Ong SH, Foong KWC. A robust global and local mixture distance based non-rigid point set registration. Pattern Recognition. 2015;48(1):156–173. 10.1016/j.patcog.2014.06.017

