# Correction: Robust CPD Algorithm for Non-Rigid Point Set Registration Based on Structure Information

**DOI:** 10.1371/journal.pone.0204544

**Published:** 2018-09-20

**Authors:** Lei Peng, Guangyao Li, Mang Xiao, Li Xie

A month before publication of this *PLOS ONE* article [1], the authors published a closely related study in the *Journal of Electronics and Information Technology* (*JEIT*), titled, “Non-rigid Point Set Registration Based on Neighbor Structure and Gaussian Mixture Models” [2]. This second article was not cited or discussed in the *PLOS ONE* article.

The authors provide the following clarification regarding the relationship between work reported in these articles:

The *JEIT* article described how to use neighbor structure information and Gaussian Mixture Models (GMM) to process the problem of point set registration [2]. The *PLOS ONE* article described the improvement of Coherent Point Drift by adaptive proportion of each GMM component and automatic outlier processing [1]. The implementation methods and the programming codes are different in the two articles, as are the membership probabilities of GMM components, the methods of calculating parameter “v”, and the experimental results.

The authors also note that there is some overlap in text and equations reported in these two articles, for which the *JEIT* article was not appropriately cited. These are described in the following table:

**Table:** 

	in the *JEIT* article [2]	in the *PLOS ONE* article [1]
1	Paragraph 1 of Section 2	Paragraph 1 of Section 2.1 Structure descriptor.
2	Paragraph 1 of Section 3	Paragraph 2 of Section 1.1 Coherent Point Drift algorithm.
3	Paragraph 2 of Section 4	Paragraph 3 of Section 2.2 Outliers processing and the EM solution.
4	Eq (1)	Eq (7)
5	Eq (7)	Eq (3)
6	Eq (8)	Eq (10)
7	Eq (10)	Eq (9)
8	Eq (12)	Eq (12)
9	Eq (13)	Eq (13)
10	Eq (14)	Eq (14)

As noted above, there are 10 items of overlap, all related to the Methods. The first item and the fourth item discuss the Shape Context [3] used to represent the structure information of the point set. The second and from the fifth to the eighth items describe the Gaussian GMM [4] which is used to process the problem of point set registration. The third, the ninth and the tenth items describe the calculation of the outlier ratio.
